# Factors associated with home delivery in rural Sindh, Pakistan: results from the global network birth registry

**DOI:** 10.1186/s12884-022-04516-2

**Published:** 2022-03-08

**Authors:** Afreen Sadia, Shafaq Mahmood, Farnaz Naqvi, Seemab Naqvi, Zahid Soomro, Sarah Saleem

**Affiliations:** Department of Community Health Sciences, Aga Khan University, PO Box 3500, Stadium Road, Karachi, 74800 Pakistan

**Keywords:** Home delivery, Rural Populations, Maternal Factors, Pakistan, Global Network

## Abstract

**Background:**

According to global estimates for 2017, nearly 295,000 maternal deaths occurred worldwide. Thus, approximately 810 women die every day due to pregnancy-related complications. This burden of maternal deaths in LMICs is primarily due to poor healthcare service utilization, as indicated by relatively low rates of institutional deliveries and skilled-birth attendance (SBA). We conducted this study with an aim to assess the factors associated with home delivery and its subsequent effect on the pregnancy outcome in rural Sindh, Pakistan.

**Methods:**

Data for this study were taken from The Global Network’s Maternal Newborn Health Registry (MNHR), which is a prospective, population-based observational cohort study. Registry data for 2018–2019 for District Thatta, Pakistan was retrieved for the analysis. Multivariable logistic regression models were used to determine the effect of each independent variable on the place of delivery by including all predictors and covariates. Results of the regression analyses are presented with crude odds ratios (OR) and adjusted odds ratios (aOR) with 95% confidence intervals (CIs).

**Results:**

A total of 4649 women were included in the study, of these, 1286 (27.7%) women had delivered at home. Of those who delivered at home, a larger proportion was illiterate (90%), had a BMI of less than 18.5 kg/m^2^ (26.0%), had parity of 3 or more (48.1%), and had a history of pregnancy loss as compared to women who had institutional delivery. In addition, two-thirds of women (63.4%) who had delivered at home had less than 4 ANC visits, whereas 15.6% did not receive any ANC. On multivariable logistic regression we found that home delivery was significantly associated with being illiterate (aOR = 1.60; [95% CI: 1.34, 2.04]), having high parity (aOR = 1.91; [95% CI: 1.58, 2.32]), and no ANC visit (aOR = 14.8; [95% CI: 10.2, 21.5]).

**Conclusions:**

More than a quarter of our study sample women delivered at home. These women were illiterate, multiparous, and did not receive antenatal care during pregnancy. It is essential to conduct extensive educational interventions for the women and their family members regarding the potential benefits of delivering in a safe and skilled environment. Moreover, the provision of comprehensive and quality antenatal care should be ensured as it improves the mothers' health-seeking behavior and helps them make informed decisions about their health and well-being.

## Background

According to global estimates for 2017, nearly 295,000 maternal deaths occurred worldwide that approximates to 810 women dying every day due to pregnancy-induced complications. A huge disparity was seen in the global burden of maternal mortality with more than 90% of deaths occurring in low-and middle-income countries (LMICs). Also, Sub-Saharan Africa and Southern Asia are together responsible for more than three-fourths of the estimated maternal mortality in 2017 [1]. This burden of maternal deaths in LMICs is primarily due to poor healthcare service utilization, as indicated by relatively low rates of institutional deliveries and skilled-birth attendance (SBA) [2, 3].

During the last decade, Pakistan has witnessed a gradual decline in its maternal mortality ratio (MMR) from 276 to 186 deaths per 100,000 live births [4]. However, it is still far away from reaching the target of 70 per 100,000 live births by 2030 as mentioned in Sustainable Development Goal (SDG) 3 [4]. Most maternal deaths occur due to a complex interplay of various social, economic, cultural, and health-system-related factors. These may include reduced accessibility to reproductive healthcare services, delayed health-seeking behaviors, lack of a skilled workforce, inadequate supplies and equipment, and poor referral services [5].

In LMICs, quality pregnancy and delivery care is largely provided by the private sector and mostly in urban areas, but it comes with a cost [6]. Public sector hospitals are overburdened, understaffed, and lack quality. With more emphasis on facility-based delivery by the experts, more women are now approaching hospitals in LMICs for delivery care, and this change is observed in rural areas as well.

In Pakistan, in large urban areas, 81% of deliveries occur in a facility, while in rural areas this proportion drops to 50% [4]. This urban–rural difference for the place of delivery is highest in Sindh with 42% of childbirths occurring at home in rural areas as compared to 28% in urban areas [4]. In our study site in district Thatta Sindh, basic and comprehensive emergency obstetric and newborn care (B- and C- EmONC) services are offered at different tiers of the government healthcare system, which is also supported by several small maternity homes and private providers. In the public sector, the primary level facilities include basic health units (BHUs), and rural health centers (RHCs) that provide B-EmONC services, whereas, the 24-h C-EmONC services are provided by Taluka (sub-district) and district level hospitals that act as secondary level facilities [7, 8]. In rural areas, because of poverty and long distances to health facilities, women still go for home delivery by an unskilled birth attendant [9, 10] which can result in complications such as stillbirth, post-partum hemorrhage, neonatal tetanus, retained products of conception, and perineal injuries [9–11]. Although there is mixed evidence available from LMICs regarding institutional births, deliveries that take place at health care facilities with a better enabling environment and good quality of care have been linked to improved survival of both the mother and the baby [12–16].

Women’s preference for home delivery is mostly influenced by their previous obstetrical experience, distance to the facility, socio-economic status, education level, order of birth, limited role in decision making, and easy access to traditional birth attendants (TBAs) [17–19]. Few studies in Pakistan have explored the factors related to women’s preference for home vs. facility-based delivery [20]. However, these factors are not well understood in rural settings where the magnitude of the problem is even higher [21]. We conducted this study with an objective to assess the factors associated with home delivery and its subsequent effect on the pregnancy outcome in district Thatta rural Sindh, Pakistan.

### Methodology

#### Study design and population

Data for this study were taken from The Global Network’s Maternal Newborn Health Registry (MNHR), which is a prospective, population-based observational study based on a birth registry that has been maintained for more than 15 years [22]. Registry data for 2018–2019 for District Thatta, Pakistan were retrieved for this analysis from the local site. All married women aged 18 years and above who gave birth in the previous six months were considered eligible. A total of 4649 women were included in the analysis and were stratified into women who gave birth at home and those who gave birth at a health facility.

#### Data collection procedure

This study was undertaken as part of the Global Network for Women and Children’s Health Research (Global Network), a multi-country research network funded by the US National Institute of Child Health and Human Development (NICHD). The MNHR is a prospective, population-based observational study that includes all pregnant women, their newborns, and their outcomes in defined geographic communities (clusters). In these clusters, there are approximately 300 to 500 births annually. There are currently 8–10 clusters at each of the Global Network sites in western Kenya, Zambia (Kafue and Chongwe), the Democratic Republic of the Congo (DRC) (North and South Ubangi Province), Pakistan (Thatta in Sindh Provence), India (Belagavi and Nagpur), Guatemala (Chimaltenango) and Bangladesh (District Tangail). The MNHR was initiated at each of the study sites between 2008 and 2009, except for the DRC, which joined the Global Network in 2014, and the Bangladesh site which joined the Global Network in 2018. The objective of the MNHR study is to enroll pregnant women by 20 weeks gestation and to obtain data on pregnancy outcomes for all deliveries that take place within the community. Each community employs registry administrators (RAs) who are paid community health workers or nurses recruited from the local communities. Every RA identifies and tracks these pregnancies and their outcomes in coordination with community elders, birth attendants, and other healthcare workers. Follow-up visits are conducted after delivery and at 42-days post-partum. During the follow-up visit, in addition to pregnancy outcome, questions related to ANC visits are asked. Moreover, the provision of specific preventive care services such as the usage of vitamins/iron, tetanus toxoid (TT) vaccine, and HIV testing are also captured as binary responses. The research administrator at each site ensures the completeness and accuracy of the data collected at each site [23, 22].

#### Outcomes and independent variables

We used registry data of 2018–2019 for Pakistan for our study purpose. Our study outcome was the place of delivery categorized as home or facility delivery. Factors related to the place of delivery were categorized into the following: 1) Socio-demographic including maternal age, educational status, socioeconomic status, 2) Maternal factors including BMI, hemoglobin, parity, the outcome of last pregnancy, and history of previous C-sections, 3) Pregnancy and delivery care including the number of ANC visits, iron and calcium supplements, maternal tetanus vaccination, maternal complications, obstetrics complications, and birth outcomes.

#### Operational definitions

##### Maternal education

We have categorized maternal education into illiterate and literate. Illiterate refers to all those women having no formal schooling and were not able to read or write, whereas women having any schooling and were able to read or write were categorized as literate.

##### Socioeconomic status

Composite scores were calculated based on assets and house ownership then categorized on basis of quartiles (lower, middle, and high).

##### Suspected reproductive tract infection (RTI)

Defined as an infection of the genital tract in which fever is present with pelvic pain, abnormal vaginal discharge/ presence of pus, abnormal smell / foul odor of discharge, and/or delay in the reduction of the size of the uterus (< 2 cm per day in the first 8 days).

##### Pregnancy and delivery complications

It includes obstructed labour/Prolonged labour/failure to Progress in labor /antepartum hemorrhage/ / hypertension, pre-eclampsia, eclampsia, postpartum hemorrhage/ and suspected RTI.

##### Pregnancy loss

We have considered stillbirth (death of fetus > 20 weeks) as pregnancy loss.

#### Statistical methods

SPSS V.19.0 (SPSS Inc, Chicago, Illinois, USA) was used for the analysis. Frequencies and proportions were reported for all the independent factors. We have used the Chi-square test to assess the frequency distribution and the relationship of independent factors with the place of delivery. The strength of association between the outcome variable and independent variables (covariates) is expressed by calculating the odds ratio (OR) and their 95% confidence interval. Univariate logistic regression was applied and variables with a *p*-value of < 0.2 were included in multivariate analysis using forward stepwise multiple logistic regression techniques to evaluate the independent effect of each variable on the place of delivery by controlling the effect of other variables. Finally, multivariable logistic regression models were developed to determine the effect of each independent variable on the place of delivery by including all predictors and covariates. Results of the regression analyses are presented with crude odds ratios (OR) and adjusted odds ratios (aOR) with 95% confidence intervals (CIs).

#### Ethical approval

The study was approved by the Ethical Review Committee (ERC) of Aga Khan University. All women provided informed consent for participation in the study including data collection and the follow-up visits at the time of data collection by MNHR research assistants.

## Results

### Socio-demographic characteristics of the study participants

A total of 4649 women were included in this study, of these, 1286 (27.7%) women had delivered at home. In both the groups, almost all the women were in the age group of 20–35 years. A larger proportion of women (89.9%) who delivered at home were illiterate having no formal education as compared to those who delivered at a facility (80.4%). No major socioeconomic differences were observed between the two groups (Table 1).

**Table 1:** Socio-demographic and maternal factors of the study participants *n* = 4649

Variables	Women delivering at home		Women delivering at a facility	
	***N* = 1286**		***N* = 3363**	
	**n**	**%**	**n**	**%**
***Socio-demographic Factors***				
**Maternal age (years)**				
< 20	32	2.50%	118	3.50%
20–35	1149	89.30%	3076	91.50%
> 36	105	8.20%	169	5.00%
**Maternal education**^a^				
Illiterate	1156	89.90%	2703	80.40%
Literate	130	10.10%	660	19.60%
**Socioeconomic status**^**b**^				
Lower	591	46.20%	1599	47.50%
Middle	684	53.00%	1726	51.30%
High	11	0.80%	38	1.20%
***Maternal Factors***				
**BMI (kg/m**^**2**^**)**				
< 18.5	330	25.80%	693	20.70%
18.5–23	741	57.80%	1861	55.60%
> 23	210	16.40%	792	23.70%
**Maternal hemoglobin (gm/dl) *n* = 4623**				
< 8	153	12.00%	363	10.80%
8–10	688	54.00%	1599	47.80%
10.1 and above	433	34.00%	1386	41.40%
**Parity**				
0	222	17.30%	900	26.80%
1–2	446	34.70%	1214	36.10%
3 and above	618	48.10%	1249	37.10%
**Outcome of last pregnancy**^**c**^				
Pregnancy loss	247	19.20%	961	28.60%
Live Birth	1039	80.80%	2402	71.40%
**History of C-sections**				
No	1239	96.30%	2992	89.00%
Yes	47	3.70%	371	11.00%

^a^Illiterate includes women with no schooling and Literate includes primary, secondary, higher secondary and above

^b^Composite scores were calculated based on assets and house ownership and then categorized on basis of quartiles

^**c**^Pregnancy loss includes stillbirths and miscarriages

### Pregnancy and delivery care factors of the study participants

A higher proportion of women (26%) who delivered at home had a BMI < 18.5 kg/m^2^ as compared to the institutionally delivered women (20.7%). Around 17.3% of women who delivered at home and 26.8% who delivered at the facility were nulliparous. Nearly half of the home-delivered women (48.1%) had parity of 3 or more as compared to 37% who delivered at a hospital. The history of miscarriage or stillbirth was more common in institutionally delivered women (28.6%) as compared to home-based births (19%).

For the most recent delivery, the proportion of women who had not received any ANC was higher (15.6%) among women who delivered at home as compared to the facility-based births (1.5%). Moreover, a vast majority of women (63.4%) who delivered at home were unable to meet the minimum criteria of 4 ANC visits as compared to the institutional deliveries (44.9%) (Table 2). In addition, the frequency of tetanus vaccination was also higher for women who had facility-based delivery (56%) as compared to the women who had home births (33%). Approximately 30% of the women delivering at home had gestational age < 37 weeks as compared to those delivering at a facility i.e., 28.9%. Among home deliveries, 4.8% did not have a live birth as compared to 4.6% among facility-based births. (Table 2).

**Table 2:** Pregnancy and delivery care factors of recent pregnancy *n* = 4649

Variables	Women delivering at home		Women delivering at facility	
	***N* = 1286**		***N* = 3363**	
	**n**	**%**	**n**	**%**
**Received ANC during pregnancy**				
No ANC	201	15.60%	51	1.50%
< 4 visits	815	63.40%	1509	44.90%
4 or more visits	270	21.00%	1803	53.60%
**Maternal tetanus vaccinations**				
No	860	66.90%	1475	43.90%
Yes	426	33.10%	1888	56.10%
**Iron supplementation**				
No	534	41.50%	706	21.00%
Yes	752	58.50%	2657	79.00%
**Calcium supplementation**				
No	493	38.30%	576	17.10%
Yes	793	61.70%	2787	82.90%
**Gestational age at delivery (weeks)**				
< 37	268	30%	936	28.90%
> 37	625	70%	2304	71.10%
**Obstructed labour/prolonged labour**				
**/failure to progress**				
No	1260	98%	3135	93.20%
Yes	26	2.00%	228	6.80%
**Obstetric hemorrhage**				
No	1266	98.40%	3206	95.30%
Yes	20	1.60%	157	4.70%
**Hypertension/preeclampsia/eclampsia**				
No	1277	99.30%	3200	95.20%
Yes	9	0.70%	163	4.80%
**Suspected reproductive tract infection (RTI)**^**a**^				
No	1272	98.90%	3212	95.50%
Yes	14	1.10%	151	4.50%
**Birth Outcomes of recent pregnancy**^**b**^				
Live Birth	850	66.10%	3093	91.90%
Not a live birth	43	4.8%	148	4.6%
**Birth weight (gm)**				
Normal weight (≥ 2500)	668	74.80%	2490	76.90%
Low birth weight (< 2500)	225	25.20%	750	23.10%

^a^Defined as an infection of the genital tract in which fever is present with pelvic pain, abnormal vaginal discharge/ presence of pus, abnormal smell / foul odor of discharge, and/or delay in the reduction of the size of the uterus (< 2 cm per day in the first 8 days)

^b^Not a live birth includes stillbirths

### Regression analysis of factors associated with place of delivery (home vs. Facility)

In univariate analysis, older maternal age, illiteracy, higher parity, and live birth outcome of last pregnancy were significantly associated with the place of delivery (Table 3). In multivariable logistic model,after adjusting for age, education, BMI, parity and ANC visits, we found, that women who were illiterate (aOR = 1.60; [95% CI: 1.34, 2.04]), having BMI of < 18 (kg/m^2^) and > 23 (kg/m^2^) (aOR = 1.21; [95% CI: 1.03, 2.42] and aOR = 1.77; [95% CI: 1.64, 2.92, respectively]), and without any ANC [95% CI: 10.2, 21.5] had greater odds of delivering at home as compared to their counterparts (Table 4).

**Table 3:** Univariate Logistic Regression of Socio-demographic, Maternal, Pregnancy and Delivery care factors associated with home delivery, crude odds ratios and 95% CI (*n* = 4649)

Variable	Crude Odds Ratio (95% CI)	*P*-value
***Socio-demographic Factors***		
**Maternal age (years)**		
< 20	ref	
20–35	1.37 (0.92, 2.04)	0.113
> 35	2.29 (1.44,3.63)	< 0.001
**Maternal Education**		
Illiterate	2.17(1.77, 2.65)	< 0.001
Literate	ref	
***Maternal Factors***		
**BMI (kg/m**^**2**^**)**		
18.5–23	ref	
< 18.5	1.19 (1.02, 1.39)	0.025
> 23	0.66 (0.56, 0.79)	< 0.001
**Maternal Hemoglobin (gm/dl)**		
< 8	1.34 (1.08, 1.67)	0.007
8–10	1.37 (1.19, 1.58)	0.002
10.1 and above	ref	
**Parity**		
0	ref	
1–2	1.49 (1.24, 1.78)	< 0.001
3 and above	2.00 (1.68, 2.39)	< 0.001
**Outcome of Last pregnancy**		
Pregnancy Loss	ref	
live birth	1.68 (1.44, 1.97)	< 0.001
**History of C-sections**		
No	3.26 (2.39, 4.45)	0.002
Yes	ref	
***Pregnancy and delivery care Factors***		
**Received ANC during pregnancy**		
No ANC	26.39 (18.87,36.71)	< 0.001
< 4 visits	3.61 (3.09, 4.20)	< 0.001
4 or more visits	ref	
**Maternal tetanus vaccination**		
No	2.58 (2.26, 2.96)	< 0.001
Yes	ref	
**Iron supplementation**		
No	2.67 (2.33, 3.07)	< 0.001
Yes	ref	
**Calcium supplementation**		
No	3.01 (2.61, 3.47)	< 0.001
Yes	ref	
**Gestational age at delivery(weeks)**		
< 37	1.06 (0.89,1.24)	0.513
> 37	ref	
**Complications in pregnancy and after delivery**^**a**^		
Yes	ref	
No	0.28(0.19,0.32)	< 0.001
**Birth Outcome of recent pregnancy**^**b**^		
Live birth	ref	
Not a live Birth	4.89(4.17,5.73)	< 0.001
**Birth weight (gm)**		
Normal Weight (≥ 2500)	ref	
Low birth weight (< 2500)	1.19(0.94,1.32)	0.2

^a^Obstructed labour/Prolonged labour/failure to Progress/antepartum hemorrhage/ postpartum hemorrhage/ hypertension, pre-eclampsia, eclampsia and severe infection

^b^Not a live birth includes stillbirths

**Table 4:** Multivariable Logistic Regression of Socio-demographic, Maternal, Pregnancy and delivery care Factors associated with home delivery, adjusted odds ratios and 95% CI (*n* = 4649)

Variable	Adjusted OR (95% CI)	*p*-value
***Socio-demographic Factors***		
**Maternal age (years)**		
< 20	ref	
20–35	1.01 (0.60, 1.53)	0.67
> 35	1.30 (0.79, 2.14)	0.62
**Maternal Education**		
Illiterate	1.60(1.34, 2.04)	0.02
Literate	ref	
***Maternal Factors***		
**BMI (kg/m**^**2**^**)**		
18.5–23	ref	
< 18.5	1.21 (1.03,2.42)	< 0.002
> 23	1.77 (1.64,2.92)	< 0.002
**Maternal Hemoglobin (gm/dl)**		
< 8	1.04 (0.88,1.31)	0.03
8–10	1.18 (1.02,1.36)	< 0.001
10.1 and above	ref	
**Parity**		
0	ref	
1–2	1.67 (1.37,2.63)	0.06
3 and above	1.91 (1.58, 2.32)	0.02
**History of C-sections**		
No	2.97 (2.15,4.11)	< 0.02
Yes	ref	
***Pregnancy and delivery care Factors***		
**Received ANC during pregnancy**		
No ANC	14.8 (10.2,21.5)	< 0.001
< 4 visits	2.73 (2.32,3.2)	< 0.001
4 or more visits	ref	
**Calcium supplementation**		
No	1.48 (1.24,1.76)	0.03
Yes	ref	
**Complications in pregnancy and after delivery**^**a**^		
Yes	ref	
No	0.34 (0.12,0.43)	< 0.001

^a^Obstructed labour/Prolonged labour/failure to Progress/antepartum hemorrhage/ postpartum hemorrhage/ hypertension, pre-eclampsia, eclampsia and severe infection

## Discussion

In our study sample, more than a quarter (27.7%) of pregnant women had delivered at home. A larger proportion of women (89.9%) who delivered at home were illiterate having no formal education. Older age group, being illiterate, having high parity, having no ANC visits were the factors which were significantly associated with home delivery. Also, those who delivered at home were twice more at odds of developing some form of complication either natally, during delivery, or in the post-partum period.

Although the institutional delivery rates have increased steadily in Pakistan over the past two decades (from 13% in 1991 to 66% in 2018), more than a third of women in rural areas still deliver at home. With more emphasis on facility-based delivery by the experts, more women are now approaching hospitals in LMICs for delivery care. However, the poorly prepared health system is not yet ready to take the brunt of ever-increasing numbers of women approaching the hospitals, and quality of care has become a major issue in providing optimal care to women and their newborns [6]. Despite the availability of health centers in almost all administrative areas of the province, previous studies have also depicted poor utilization and access to quality maternal care services. According to the recent Pakistan Demographic and Health Survey (PDHS 2017–18), about 1 in 3 births in Pakistan take place at home, and this proportion is much higher in the rural areas (41%) as compared to the urban areas (19%) [24]. Literature from other regions of the world has also established a higher rate of obstetrical complications among rural women [25, 26]. It has been found that rural women have a 9 times increased risk of severe morbidity and mortality following childbirth [25]. Although there are several clinical and social determinants of this huge rural–urban disparity in maternal health, the lack of professional delivery care is one of the key factors responsible for this inequality. Hence, an important step towards improving maternal and child health outcomes is to increase the coverage of skilled delivery care for rural women [26].

The literature has established a positive association between maternal age and place of delivery [27–29]. Young teenage mothers are more likely to deliver at a health care facility as compared to older mothers. One possible explanation for this is the gradual decline in the perceived complexity of pregnancy and childbirth complications with rising age. [28] Parveen Z et al. in their secondary analysis of PDHS 2012–13 reported 11 times higher odds of home delivery in women above 34 years of age [28]. Similarly, in our study women above 35 years of age were twice as likely to deliver at home as compared to their younger counterparts. Experience of successful deliveries conducted at home might serve as a future determinant of home-based delivery in older women [30]. Also, older age females are more inclined towards traditional methods and are less likely to use modern healthcare facilities [31].

Another factor influencing the delivery place is the parity of the mother. In our study, we found nearly half (48%) of the multiparous women delivering at home as compared to the nulliparous women. Similar results were reported by a study in Nepal where a high prevalence of home deliveries was found in multiparous women [29]. It is believed that since nulliparous females do not have any prior experience of childbirth and are more apprehensive about their delivery, thus they are more likely to deliver at an institutional setting as compared to the multiparous women who have had their past deliveries conducted at home [32]. Therefore, older females with higher parity should be specifically targeted during educational intervention programs to raise their awareness regarding the importance of institutional deliveries.

Furthermore, higher educational attainment by both the mother and the father has proved to be important predictors of hospital delivery [33]. A person with better educational status is more likely to understand the health education messages that ultimately lead to improved health decisions [34]. Our study also found that uneducated mothers were more inclined towards home-based delivery. These findings are consistent with the previous studies conducted in low-income countries where women with better educational status were 9 times more likely to have an institutional delivery as compared to the less educated mothers [33, 35]. It has been postulated that a woman becomes more influential in the decision-making process with higher educational attainment and thus able to make better choices for herself and her baby [35].

According to the findings of a qualitative survey conducted in rural Sindh, poor quality of health care services and fear of operative delivery also inhibited the mothers from having institution-based deliveries. Some of the females believe that doctors conduct unnecessary C-sections to earn money, hence they prefer to deliver at home. Moreover, some females also complained about the non-supportive and non-empathetic behavior of doctors with the mother during childbirth, hence they are more comfortable having their deliveries with traditional birth attendants (TBA) who spend more time listening to their concerns and problems [36].

ANC utilization during pregnancy is another important predictor of institutional delivery. It is often considered as the first point of contact for many women to gain information regarding their pregnancy and possible complications they may encounter during childbirth. Tsegay et al. established a positive correlation between ANC attendance and hospital delivery [37]. Likewise, studies from other LMICs have also reported similar findings [17, 29, 38]. In our study, women who did not avail of any ANC services were fourteen times more likely to deliver at home as compared to the women with four or more ANC visits. The ANC coverage in Pakistan has improved much in the past few years, but a vast majority of females do not meet the minimum WHO criteria of eight ANC visits [24]. In our study, around 67% of the women had no tetanus vaccination which is also reflected by poor ANC during the pregnancy. These results are in line with a study done by Yaya et al. in which women who had attended at least four ANC visits had higher odds of having tetanus immunization [39]. Failing to optimize ANC services for rural women is a potential missed opportunity to reduce adverse health outcomes associated with unsafe childbirth.

In this study, we also found that women who delivered at home had more complications as compared to their counterparts. Childbirth is often considered to be a normal phenomenon by many rural females, and in the absence of complications in previous pregnancies, they usually wish to deliver at their homes. A qualitative survey conducted in rural settings of a lower-income country reported that even those females who lived near a healthcare facility did not consider hospital delivery essential when the perceived risk of labor was low [40]. Also, in another study home deliveries were associated with increased maternal morbidity especially the third stage complications [41].

The findings of this study have shed light on some of the key factors that serve as a barrier to safe and skilled delivery in an institutional setting. We found that the odds of having a home delivery were significantly higher among less-educated females, with higher parity, and with low ANC attendance. Considering the above findings, stakeholders and policymakers should develop effective strategies that could overcome the barriers to safe and skilled delivery of care, as identified by this study. For example, educating mothers with a previous history of home delivery could be useful in shaping their future decision by improving their understanding of the potential benefits associated with hospital-based delivery [20]. Also, radical steps should be taken to reduce gender disparity in education, particularly in rural areas, where females are discouraged to acquire basic education. It is implied that an educated female is more receptive to educational messages and is better able to make an informed decision about her health [34]. Lastly, the government should ensure timely and adequate contact of females with the healthcare system through the provision of quality ANC services as it is positively associated with improved maternal and newborn outcomes [37].

With more emphasis on facility-based delivery by the experts, more women are now approaching hospitals in LMICs for delivery care. However, the poorly prepared health system is not ready to take the brunt of ever-increasing numbers of women approaching the hospitals, and quality of care has become a major issue in providing optimal care to women and their newborns [6]. Despite the availability of health centers in almost all administrative areas of the province, previous studies have depicted poor utilization and access to quality maternal care services.

A few limitations of the study need to be noted. As this was secondary data analysis, our study could not explore many of the health system-related factors such as quality and cost of services, availability of drugs and equipment, and training of health care providers. In the SES variable, we don’t have data on household income therefore results should be interpreted with caution. Further studies should be conducted in the future to objectively measure healthcare-related factors that may serve as a barrier to institutional delivery in Pakistan. In addition, there was also a chance of recall bias as some of the prenatal and intrapartum information was collected from the mothers after their delivery.

## Conclusion

In our study, nearly one-third of the deliveries took place in home settings. Older age, multiparous, less educated females, and those who did not receive antenatal care during their pregnancy were more likely to deliver at home. This study has provided insight into various socio-demographic and maternal factors that are associated with a home as a place of delivery. It is essential to conduct extensive educational interventions for the mothers and their family members regarding the potential benefits of delivering in a safe and skilled environment. Moreover, the provision of comprehensive and quality antenatal care should be ensured as it improves the mothers' health-seeking behavior and helps them make informed decisions about their health and well-being.

## Data Availability

The datasets that were analyzed can be access at NICHD data repository (NDASH): https://dash.nichd.nih.gov/

## Abbreviations

SBA: Skilled-birth attendance
MNHR: Maternal Newborn Health Registry
LMICs: Low-and middle-income countries
TBAs: Traditional birth attendants
BMI: Body mass index
